# Cationic Nano-Lipidic Carrier Mediated Ocular Delivery of Triamcinolone Acetonide: A Preclinical Investigation in the Management of Uveitis

**DOI:** 10.3390/life13041057

**Published:** 2023-04-20

**Authors:** Pradip Nirbhavane, Laxmi Moksha, Gajanand Sharma, Thirumurthy Velpandian, Bhupinder Singh, O. P. Katare

**Affiliations:** 1 UGC-Centre of Advanced Study, Division of Pharmaceutics, University Institute of Pharmaceutical Sciences (UIPS), Panjab University, Chandigarh 160014, India; 2 Dr. Rajendra Prasad Centre for Ophthalmic Sciences, Ocular Pharmacology and Pharmacy Division, All India Institute of Medical Sciences, New Delhi 110029, India

**Keywords:** uveitis, nano-lipoidal, triamcinolone acetonide, lipopolysaccharide, aqueous humor

## Abstract

The current study was undertaken to evaluate the efficacy of a novel nano-lipoidal eye drop formulation of triamcinolone acetonide (TA) for the topical treatment of uveitis. The triamcinolone acetonide-loaded nanostructured lipid carriers (cTA-NLC) were developed by employing ‘hot microemulsion method’ using biocompatible lipids, which exhibited a sustained release nature and enhanced efficacy when evaluated in vitro. The in vivo efficacy of this developed formulation was tested on Wistar rats, and a single-dose pharmacokinetic study was conducted in rabbits. The eyes of animals were examined for any signs of inflammation using the ‘Slit-lamp microscopic’ method. The aqueous humor collected from the sacrificed rats was tested for total protein count and cell count. The total protein count was determined using BSA assay method, while the total cell count was determined by Neubaur’s hemocytometer method. The results showed that the cTA-NLC formulation had negligible signs of inflammation, with a clinical score of uveitis 0.82 ± 0.166, which is much less than control/untreated (3.80 ± 0.3) and free drug suspension (2.66 ± 0.405). The total cell count was also found to be significantly low for cTA-NLC (8.73 ± 1.79 × 10^5^) as compared to control (52.4 ± 7.71 × 10^5^) and free drug suspension (30.13 ± 3.021 × 10^5^). Conclusively, the animal studies conducted showed that our developed formulation holds the potential for effective management of uveitis.

## 1. Introduction

Intraocular inflammation, also called as ‘uveitis’, is one of the most common causes of visual loss. It accounts for about 10–15% cases of total blindness in the developed world and up to 25% in the developing world [1]. It is a group of disorders with more than 30 diseases characterized by inflammation inside the eye. It should not be confused with ocular surface inflammation (cornea), i.e., keratitis [2]. The goal of the treatment of uveitis is based on controlling the ocular inflammation, preventing impairment of visual acuity, and improving the patient’s quality of life [3]. According to the etiological cause, uveitis may be classified as infectious and non-infectious. The treatment modalities for the non-infectious uveitis are based on (a) topical corticosteroids (especially for anterior uveitis), (b) systemic low-dose corticosteroids, (c) intravitreal corticosteroids for severe patients with posterior uveitis, (d) systemic immunomodulators (T-cell inhibitors) and (e) biologics (e.g., infliximab) [4]. Topical corticosteroids are the mainstay therapy for treating non-infectious uveitis. However, they are usually associated with adverse effects such as cataracts, corneal-epithelium toxicity and steroid-induced glaucoma [5]. Furthermore, the intravitreal or subtenon injections of steroids such as TA can have various adverse events, such as elevated intraocular pressure, cataract formation and also injection-related complications such as endophthalmitis, secondary ocular inflammation and retinal detachment [6,7]. The topical steroidal eye drops are not fully effective, as their corneal permeation is affected due to strong defensive barriers such as a corneal epithelial barrier [8,9]. Hence, in order to improve corneal permeation and retention as well as to curb the adverse effects associated with topical steroids, the novel drug delivery system of TA was designed and developed. 

In order to target the drug at its site of action only and avoid adverse effects of conventional dosage forms, we have developed topical dosage form (i.e., eye drop formulation) of TA, which is conventionally given as an intravitreal or subtenon injection. The developed topical dosage form is based on nanostructured lipid carriers (NLC) as drug delivery vehicles. The developed dosage form has shown its in vitro anti-inflammatory efficacy at a much lower concentration (4 mg/mL), as reported in our previous publication [10]. In this manuscript, we are reporting the in vivo effects of this formulation studied in LPS-induced Endotoxin Induced Uveitis (EIU) rat models. The endotoxin-induced uveitis (EIU) rat models have been widely used for evaluating the anti-inflammatory activity of various compounds. This model is well-established now, as it was reported long back in 1980. The EIU can be generated in animals by systemically administering Lipopolysaccharide (LPS), which is able to induce inflammatory responses mostly in the anterior part of the uvea and mildly in the posterior part of the uvea. The responses and symptoms so generated mimic the pathological conditions in human acute uveitis [11,12].

The manuscript briefly discusses the characteristics of the developed formulation and its in vivo anti-inflammatory effect in EIU rat models and single-dose pharmacokinetic study of the formulation in rabbits. 

## 2. Materials and Methods

### 2.1. Chemicals and Reagents

Triamcinolone acetonide was generously provided as a gift sample by Lupin Ltd. (Pune, India). Capmul MCM C10 (Glyceryl monocaprate), Transcutol^®^P (diethylene glycol monoethyl ether), and Captex 200P (Propylene glycol dicaprate) were generously provided as free gift samples by ABITEC Corporation (Columbus, OH, USA). Lecithin was supplied as a generous gift by Lipoid GmbH (Ludwigshafen, Germany). Polysorbate 80 was purchased from Fischer Scientific Pvt Ltd. (Mumbai, India). Stearylamine was purchased from Sigma-Aldrich, St. Louis, MO, USA. LC-MS grade acetic acid (AA), formic acid (FA), acetonitrile and methanol were purchased from Merck (Darmstadt, Germany). Ultrapure water of a Milli-Q Gradient system (Millipore Corp., Bedford, MA, USA) was used for the study. All other chemicals and reagents were of analytical grade, while HPLC solvents were of HPLC grades. 

### 2.2. Preparation and Characterization of cTA-NLC

The cTA-NLC was developed using the ‘hot microemulsion’ method, as reported in our previous publication [10]. Briefly, a weighed amount of drug (40 mg) and a lipid mixture, i.e., Capmul MCM C10, lecithin (solution in dichloromethane, 100 mg/ mL), and Captex 200, were taken in a small beaker at 1:6 *w/w* of the drug: lipid mix. The drug and lipid mix were then heated at 60 °C, followed by the addition of the surfactant mixture (Smix (Transcutol P and tween 80 in 1:1 ratio)). Once a homogenous mixture was obtained, stearylamine (solution in ethanol) was added, followed by a small amount of hot water (200 mg) (50–60 °C) to yield a microemulsion. After 5 min of stirring, the microemulsion was added dropwise to cool water (10–15 °C). The above mixture was then subjected to high shear homogenization (16,000 rpm) for 10 min (Heidolph, Silent Crusher M, Germany). The homogenized mixture was left to stir at room temperature (700 rpm) (25 °C) for 3–4 h before use. 

The developed cTA-NLC formulation was characterized for its size, PDI, zeta potential, % entrapment efficiency, osmolarity and pH. The size, PDI and zeta potential of the cTA-NLC formulation were determined on the Malvern zetasizer. A diluted suspension (1:10) of the formulation was taken for this. 

FTIR spectral analysis is also one of the analytical tools for the characterization of formulation. The FTIR spectra can provide the information about the possible interaction between various chemicals. FTIR analysis was conducted to furnish information about the groups present in the formulation and also for the exploration of potential interactions. FTIR analysis of free TA, physical mixture, blank NLC and cTA-NLC were conducted to confirm the entrapment of TA within the developed NLC. FTIR analysis of samples (10–15 mg) was carried out by the KBr pellet method, using PerkinElmer Spectrum (Version 10.03.08) spectrometer operated at a resolution of 4 cm^−1^ in the range of 450–4000 cm^−1^.

### 2.3. In Vitro Evaluation

The developed formulation was evaluated for its in vitro drug release study, skin permeation study and in vitro anti-inflammatory activity [10].

#### 2.3.1. In Vitro Drug Release Study

For the estimation of drug release in vitro, a dialysis bag method was employed. Simulated tear fluid (STF, pH = 7.4): N-methyl pyrrolidone (NMP) in a ratio of 80:20 was taken as drug release medium [13,14].

#### 2.3.2. Transcorneal Permeation Study

The transcorneal permeation study was performed using freshly excised goat cornea and a Franz diffusion cell. The drug release medium was STF: NMP in a ratio of 80:20. The drug permeation profile was compared with the permeation profile of a free drug suspension (TA-suspension) [15,16].

#### 2.3.3. Anti-Inflammatory Activity

The TNF-α elisa assay was utilized to evaluate the in vitro anti-inflammatory activity of the formulation. This study was conducted in human corneal fibroblasts (HCF) cells. The inflammation was induced in the cells by LPS from *E. coli*. Different treatments such as cTA-NLC, blank-NLC, and free drug solution (TA solution) were compared for their anti-inflammatory potential [15]. 

### 2.4. In Vivo Studies

Wistar rats (male, 150–200 g body weight) were provided by the Centre Animal Facility of the All India Institute of Medical sciences, New Delhi, India. The rats had access to standard food and water ad libitum and were maintained under alternating 12 h light and dark cycle. Prior approval from the Institutional Animal Ethics Committee (IAEC No. 186/IAEC-1/2019) was obtained before conducting any animal experiments. Animals were handled in accordance, and experiments were performed as per the guidance of the ARVO statement for the Use of Animals in Ophthalmic and Vision Research (ARVO).

#### 2.4.1. Experimental Uveitis Rat Model: Clinical Examination of the Eye

The uveitis rat models were developed using endotoxin LPS from *Escherichia coli* (Sigma Aldrich, USA). Male wistar rats were used for this study. The rats were divided into 5 groups as follows: Group I: LPS only, Group II: LPS + cTA-NLC, Group III: LPS + TA-suspension, Group IV: LPS + Blank NLC, Group V: LPS + Marketed formulation (Prednisolone acetate suspension 1%, PredForte^®^). Each group comprised 6 rats. For the induction of uveitis, LPS (0.2 mL of 100 μg/mL) was injected into the footpad of the left hind paw of the rats. The treatments (formulations) were given immediately after the LPS injection, as well as thereafter at predetermined time intervals (0 h, 12 h, 24 h). After 28 h, the rats were clinically observed for signs of inflammation using a slit lamp microscope (MICRON III, Phoenix laboratories, South El Monte, CA, USA) [5,9].

#### 2.4.2. Anterior Segment Evaluation

The pathological inflammatory changes in EIU were examined using a slit lamp microscope equipped with Streampix software using Micron III rodent imaging system (Phoenix Laboratory, Phoenix, AZ, USA). At the 28 h time point after LPS+ treatment, the rats were anesthetized using ketamine (50 mg/kg) & xylazine (7 mg/kg). The pupillary constriction caused by LPS challenge was compared with the baseline and positive control group (LPS only) [17,18]. The clinical scores were also assigned after a clinical examination of the eyes. The clinical scores were based on the inflammatory signs observed in the eyes of rats, such as hypopyon, hyperemia, fibrinoid exudation in pupillary area and pupil size. The scorings are as follows: 0 = absence of any inflammation, 1 = minimum inflammation, 2 = moderate inflammation, 3 = severe inflammation with flare in anterior chamber and 4 = same clinical sign as grade 3 plus fibrinoid exudation in the pupillary area [19,20,21].

#### 2.4.3. Protocol Aqueous Humour Sampling

Aqueocentesis was performed after 28 h of LPS and treatment, after euthanizing the animals using an excess of carbon dioxide. Approximately 30 μL of aqueous humor was obtained from each eyeball by inserting a 25-gauge scalp vein needle (ADIS, Albert David Ltd., New Delhi, India) into the anterior chamber of the eye through the mid-inferior corneal-scleral limbal junction region under the operating microscope. The samples taken into the microcentrifuge tubes were then centrifuged at 7840× *g* for 1 min, in order to settle down the inflammatory cells, and the supernatant was collected for total protein analysis [22,23,24].

#### 2.4.4. Total Protein Estimation

In 5 μL of the AH sample, 300 μL of Bradford reagent was added. This mixture was then vortexed and incubated for 5 min at room temperature. After that, 10 μL of HCl was added to stop the reaction. The optical density of the above samples was then checked on Microplate Spectrophotometer (Multimode reader, Molecular Devices, CA, USA). BSA calibration curve was also made for comparison. Briefly, 1 mg of BSA was weighed and dissolved in distilled water to yield a stock solution of 100 μg/mL. The working standard solutions such as 5.2, 10.5, 21.0, 42.1 and 84.2 μg/mL were prepared from this stock solution. The above W.S samples were treated as discussed previously [17].

#### 2.4.5. Inflammatory Cell Count

Into 10 μL of AH sample, a similar volume of Turk’s stain was added. This mixture was then added to Neubaur’s hemocytometer. The cells were then counted as the average count in 4 fields/square. The total cell count was then calculated using the formula [2]:

$$Total\;cells\;in\;1\;\mu L=\frac{Average\;no.\;of\;cells\;in\;4\;squares\times D.F}{4}\;$$

#### 2.4.6. Single-Dose Pharmacokinetics of Topical TA in Rabbits

New Zealand albino rabbits were used for this experiment. The rabbits were kept in standard laboratory conditions with a 12 h light and 12 h dark cycle. The animals were maintained with standard fresh leafy food along with drinking water ad libitum. Animal ethics permission was obtained, and experimental protocols were approved by the AIIMS animal ethics committee. The rabbits were divided into 3 groups for each time point as follows: Group I: 2 h, Group II: 8 h, Group III: 24 h. Each group contained 3 rabbits. Briefly, 50 μL of cTA-NLC formulation was instilled into the left eye of each rabbit. After their respective time points, the aqueous humor sample was collected in microcentrifuge tubes and stored at −80 °C until utilized for the analysis. The analysis of these aqueous humor samples was conducted using LC-MS.

#### 2.4.7. LC-MS/MS Analysis of TA

##### Instrumentation

A triple quadrupole tandem mass spectrometer (4000 Q-Trap, AB Sciex, Foster City, CA, USA) was employed to conduct LC-MS/MS analysis. The mass spectrometer was coupled with an HPLC system (Agilent Technologies, 1260 Infinity, Santa Clara, CA, USA) comprised of the quaternary pump (G1311C), multisampler (G7167A), thermostatted column compartment (G1316A) with variable wavelength UV detector (G1314F), and online degasser. Analyst software, version 1.5.2 (AB Sciex, Foster City, CA, USA) was utilized to control the parameters of the tandem mass spectrometer, while OpenLAB control panel software (Agilent Technologies, 1260 Infinity, Santa Clara, CA, USA) was employed to control HPLC parameters.

##### Chromatographic Conditions

Chromatographic analyses were performed on a C_18_ analytical column (Purospher Star; 50 × 4.6 mm, 3.5 µm) using a gradient of mobile phases consisting of solvent A (18.2 MΩ purified water with 0.1% Acetic Acid) and solvent B (acetonitrile) pumped at a flow rate of 0.5 mL/min. TA was eluted using gradient started at the ratio of 60:40 of A:B (0–1.5 min). It was linearly increased to 30:70 for A:B (1.5–3 min) and maintained till 4 min, followed by returning to the initial conditions at 4.5–6 min. The post-run time was kept for 2 min for equilibration between each run. The total run time for the analysis of a single sample was 6 min. The injection volume for the analysis was kept as 20 µL.

##### Mass Spectrometer Conditions

The mass spectrometer was operated with an electrospray ionization (ESI) source in positive ion mode using Turbo Ion Spray source (AB Sciex, Foster City, CA, USA). The singly protonated ion of TA and sulphadimethoxine (SDM, IS) were observed at *m*/*z* 435.2 and *m*/*z* 311.0 in ESI positive ion mode. Fragmentation spectra of TA were recorded, and abundant daughter ions were selected for the analysis. Multiple reaction monitoring (MRM) mode was used for the quantification of TA using SDM as an internal standard. Quantification of TA was performed using precursor-to-product ion transition *m*/*z* 435.2/397.2 (quantifier) and *m*/*z* 435.2/415.2 (qualifier). The mass transition used for the internal standard (SDM) was *m*/*z* 311.0/156.0.

#### 2.4.8. Quantification of TA in Aqueous Humour Samples

An accurately weighed amount of TA was dissolved in pure methanol to produce a primary stock solution having a concentration of 1 mg/mL. An aliquot of 10 µL was mixed with 990 µL of pure methanol using a calibrated 1 mL Hamilton syringe to produce a working solution (10 µg/mL). The extraction solvent was freshly prepared to consist of 70% (*v*/*v*) acetonitrile with 5 ng/mL SDM (IS). From the working solution (10 µg/mL), different concentrations were produced in the range of 0.195–25 ng/mL. From these dilutions, an aliquot of 20 µL standard/sample was mixed with 100 µL of extraction solvent, vortexed for 30 s, and centrifuged at 7840× *g* for 2 min. The clear supernatant 100 µL was loaded into a 96-well plate for LC-MS/MS analysis.

## 3. Results

### 3.1. Preparation and Characterization of the Formulation

The cTA-NLC formulation was prepared by the hot microemulsion method and characterized for its particle size, zeta potential, % encapsulation efficiency and osmolarity. All the methodologies and results of these studies were described in depth in our recently published article [10]. The size of the developed formulation was found to be less than 200 nm (~199 nm). The % EE was found to be ~88%. The surface morphology (analyzed by FE-SEM) showed spherical-shaped, nanosized particles. The results of the characterization with respect to particle size, zeta potential, drug entrapment, pH and osmolarity are given in Table 1.

#### FTIR Analysis

**Figure 1:**
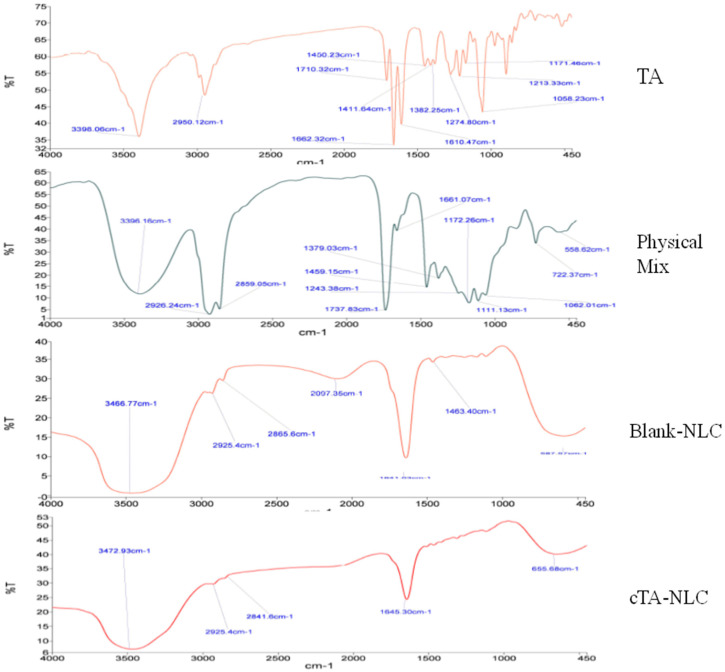
FTIR analysis of TA, Physical mixture, Blank-NLC and cTA-NLC.

FTIR spectra of TA, the physical mixture, blank NLC and cTA-NLC are shown in Figure 1. The characteristic peaks of TA were observed at 3398 cm^−1^ (O-H stretching), 2950 cm^−1^ (C-H stretching), 1710 cm^−1^ (C=O stretching), 1662 cm^−1^ (C=C stretching), 1450 cm^−1^ (C-H bending), 1274 cm^−1^ (C-O stretching). These peaks reappeared in the physical mixture with slight shifts at 3396 cm^−1^, 2959 cm^−1^, 1661 cm^−1^ and 1459 cm^−1^. In blank-NLC, the peak at 3472 is due to O-H stretching. The other small peaks at 2925 and 2865 cm^−1^ are due to strong C-H stretching and weak O-H stretching. The band at 1641 cm^−1^ is due to C=C stretching. These peaks reappeared in cTA-NLC at 3472 cm^−1^, 2925 and 2841 cm^−1^ and 1645 cm^−1^, only with a slight shifting. These results confirmed no significant interactions as well as the formation of the cTA-NLC formulation.

### 3.2. In Vitro Evaluation

The drug release study showed biphasic release behavior from the cTA-NLC formulation. In the first four hours, the drug release from formulation was observed to be rapid (around 50%). It was then followed by a slow, sustained release, i.e., at the end of 24 h, around 84% of drug release was observed. This biphasic release behavior was consistent in the transcorneal permeation study. In the first four hours, around 39% of drug permeation was observed, followed by slow permeation up to 51% at the end of 8 h [10].

In the TNF-α elisa assay, the cTA-NLC formulation exhibited the least inflammatory expressions (~1100 pg/mL) in all the treated groups (Table 2). The highest TNF-α values (~2100 pg/mL) were observed in the LPS-only treated group, followed by blank-NLC (~1600 pg/mL) and TA-solution (~1450 pg/mL), and finally cTA-NLC. Hence, it can be concluded that the developed cTA-NLC formulation is effective in reducing inflammation [10]. The results of TNF-α Elisa assay are given in Table 2.

### 3.3. In Vivo Studies in Rats

#### 3.3.1. Experimental Uveitis Rat Model: Clinical Examination of the Eye

**Figure 2:**
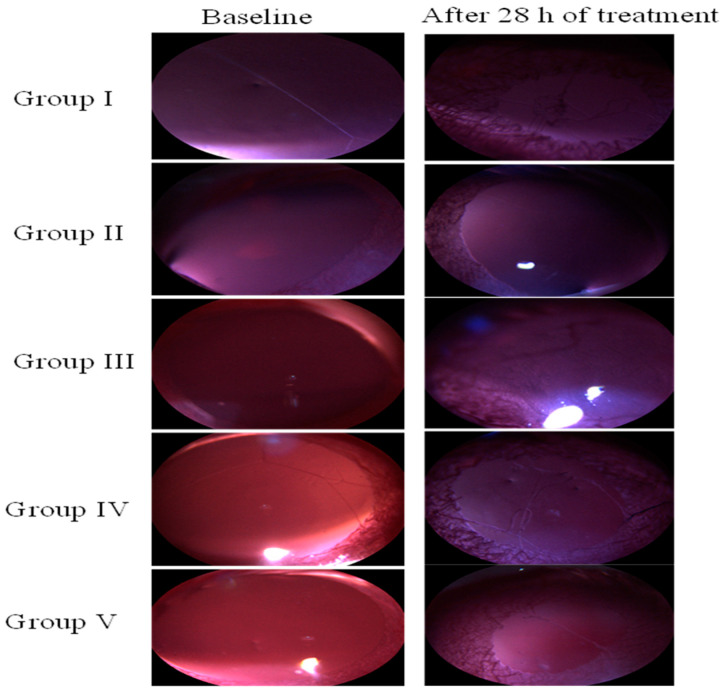
Clinical examination of eye of differently treated rat groups.

In this study, the uveitis was induced in rats by injecting LPS into the hind paw. After induction of uveitis into the rats, the clinical signs of uveitis were examined 28 h after the treatments of LPS and formulation. The images of the clinical examination of the eye are depicted in Figure 2.

**Figure 3:**
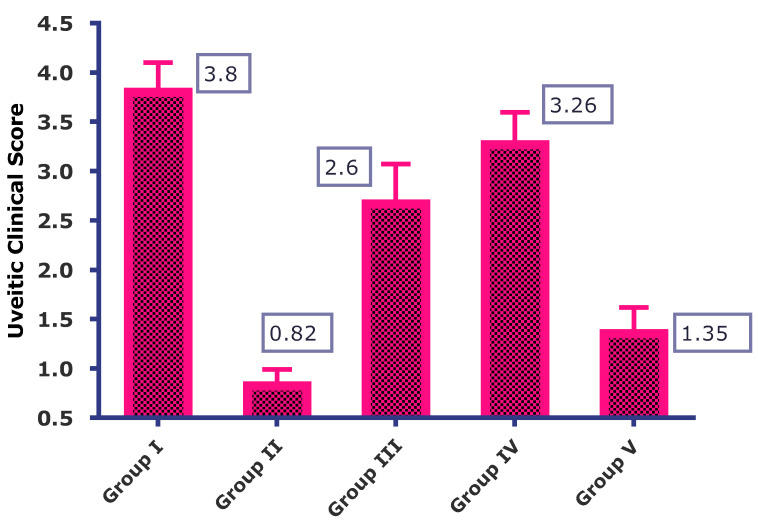
Uveitic Clinical Score of the various treatment groups.

In all the groups tested, the rats in the control group (LPS only) showed the highest level of intraocular inflammation, followed by Group IV (blank-NLC) treated group. Group I developed severe uveitis, as observed from the slit lamp images, which showed a contraction of the pupil, opacity and hyperemia. Group III also shows pupil constriction, loss of reflex and dilated blood vessels. Group II showed the least or almost negligible inflammation with no signs of hyperemia, hypopyon or pupil contraction. The clinical scores assigned to the various treatment groups are shown in Figure 3. The control group (Group I) has the highest clinical score (3.8 ± 0.3), followed by blank-NLC treated group, i.e., Group IV (3.26 ± 0.33). Group II (cTA-NLC) and Group V (Marketed formulation) have the lowest clinical score values, i.e., 0.82 ± 0.16 and 1.35 ± 0.26, respectively.

#### 3.3.2. Total Protein Count

The total protein count estimated after 28 h of treatment showed higher values in the control group, i.e., Group I (4.95 ± 0.69 mg/mL), followed by Group IV, i.e., the Blank NLC treated group (4.15 ± 0.36 mg/mL) and Group V, i.e., the marketed formulation treated group (3.70 ± 0.13 mg/mL). cTA-NLC treated rats (group II) showed the least total protein count (1.68 ± 0.23 mg/mL). The levels of total protein count in group II were significantly less than in other groups. The results of the study are given in Table 3.

#### 3.3.3. Total Cell Count

The results of the total cell count are given in Table 3. Group I showed the highest total cell count (52.4 ± 7.71 × 10^5^), followed by the blank-NLC treated group, i.e., Group IV (39.46 ± 2.71 × 10^5^). Group III (TA suspension) also showed a significantly higher total cell count (30.13 ± 3.021 × 10^5^) than Group II (8.73 ± 1.79 × 10^5^) and Group V (13.06 ± 1.52 × 10^5^).

The results of the total protein count and total cell count unanimously showed that cTA-NLC treated rats (Group II) had the least protein and cell extravasation due to less inflammation. The low level of inflammation can be attributed to the better corneal penetration and cell uptake of the cTA-NLC formulation.

#### 3.3.4. Single Dose Pharmacokinetics of TA in Rabbits/Intraocular Penetration Studies

**Figure 4:**
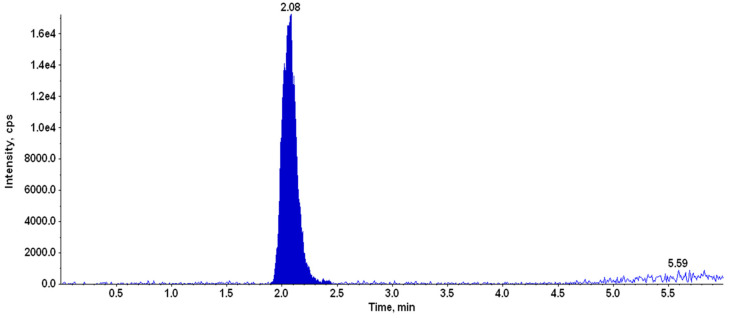
LC-MS/MS chromatogram of TA in aqueous humor (time point 2 h).

**Figure 5:**
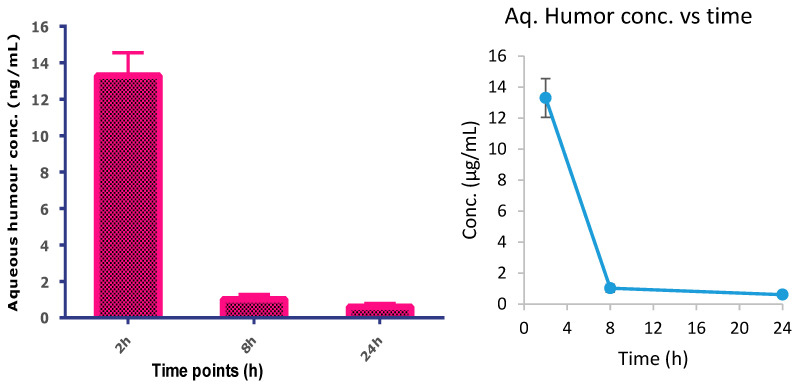
Aqueous humor concentration in rabbits at different time points (bar diagram and a scatter plot for the same).

The TA in aqueous humor was estimated with the help of LC-MS. The representative chromatogram of TA in the aqueous humor is depicted in Figure 4. The results of a single- dose pharmacokinetic study in rabbits are depicted in Figure 5. From Figure 5, it is clear that the highest drug concentration was found at 2 h time point (13.3 ± 1.25), followed by 8 h (1.02 ± 0.259) and 24 h (0.603 ± 0.188). The highest concentration of the drug was achieved in 2 h and it continuously decreases until 8 h, and it was found in negligible quantities at 24 h.

## 4. Discussion

Taking cognizance of the adverse effects associated with the marketed formulation of TA (intravitreal injection) such as the development of hypopyon, glaucoma, endophthalmitis, retinal detachment and increased risk of secondary ocular infection, as well as the limitations of the currently available eye drop formulations of other steroids (such as low ocular bioavailability) [25], we proposed to develop a novel nano-lipid carrier-based formulation of TA for topical treatment of uveitis. In ocular drug delivery systems, nano-sized particles favor higher surface-to-surface contact with biological membranes due to greater surface area for the association between cornea and conjunctiva. This will help in maintaining longer retention of NLC on the ocular mucosa, which increases its association with the tissues and active drug encapsulated within NLC [26]. Furthermore, the cornea and conjunctiva have a negative charge on their surface; thus, positively charged nanoparticles can have a huge advantage, as they can be attracted towards negatively charged ocular surface, owing to electrostatic interactions [27]. Thus, the introduction of the positive charge (through the cationic lipid) increases ionic interaction between the positively charged lipid nanocarriers and negatively charged mucus layer of the ocular surface [28]. Hence, to increase the association of the formulation with the corneal tissue, and to enhance the corneal uptake of the drug, cationic nano-lipoidal (NLC) formulation of TA was prepared for the treatment of uveitis. In our previous manuscript, we reported in detail methodology of preparation and characterization of the developed formulation. The in vitro evaluation and efficacy studies were also reported in the same manuscript. Herein, we described the in vivo efficacy of our formulation in uveitis rat models [10].

For assessing the in vivo efficacy of the developed formulation, we used rodents, more specifically Wistar rats. For the development of uveitis rat models, we used LPS for the induction of uveitis in the rats. The endotoxin-induced uveitis (EIU) model, as in the case with LPS, mimics many of the immunopathogenic mechanisms associated with human uveitis and was originally utilized as a model of anterior uveitis. Hence, this is the most suitable animal model for studying uveitis [29]. LPS creates nicks in the vessel wall and causes infiltration of cells and protein into the aqueous humor of anterior chamber. This follows a series of ocular inflammatory cascades resulting after blood-ocular barrier breach [30,31]. After induction of uveitis, the eyes of the rats were clinically examined using a slit lamp microscope for the assessment of inflammation and any other internal changes such as opacity, pupil constriction, etc. The rats were sacrificed thereafter, and aqueous humor was collected for the estimation of total protein and total cell count. Although the total protein count is not a specific sign of inflammation, it provides an idea about the amount of damage that occurs to the eye though the induction of inflammation. The inflammatory cells present in aqueous humor can serve as markers or specific signs for intraocular inflammation [32]. Earlier studies revealed that the breakdown of the blood–aqueous humor barrier causes migration of white blood cells such as leukocytes. These result into the initiation of inflammatory symptoms. We, therefore, carried out the cell count in the aqueous humor [33,34].

Based on the results of total protein count and total cell count of the studied treatment groups, it can be concluded that our formulation has better efficacy in suppressing intraocular inflammation compared to the conventional suspension of TA and marketed eye drops (Prednisolone acetate) at a much lower concentration (0.1%). This could be attributed to enhanced permeation and retention of the drug carried through the nano-lipidic carrier across the different layers of ocular tissue to the site of action.

Single-dose pharmacokinetic studies were conducted in rabbits to obtain an idea of the time at which the maximum level of the drug achieved at the site of action and how long it stays there. The results showed that the maximum available drug concentration was at 2 h time point (13.3 ± 1.25), followed by 8 h (1.02 ± 0.259) and 24 h (0.603 ± 0.188). This is probably due to the rapid absorption and distribution of TA through various ocular tissues. TA absorption is carried out mainly through the cornea and conjunctiva, with the initial distribution of the drug to the aqueous humor. However, the continuous aqueous humor replacement allows for the removal of the drug quickly from the anterior ocular chamber to the posterior chamber [35,36]. The low concentration in the aqueous humor is beneficial as it negates any possibility of dose-related adverse effects of the drug. However, the low concentration after 8 h advocates frequent administration of this eye drop, i.e., twice or thrice a day.

## 5. Conclusions

TA-loaded cationic NLC (cTA-NLC) based eye drop formulation was developed for the topical treatment of uveitis. The eye drop formulation containing cTA-NLC was tested in uveitis rats and compared with blank NLC, TA suspension and marketed formulation (Pred Forte^®^). It was observed that our formulation was able to reduce the inflammation of the uveitis significantly as compared to other treatments, as observed in the results of slit-lamp examination, total protein count and total cell count. The single-dose pharmacokinetic studies in rabbits showed that the highest concentration of the drug was achieved in the aqueous humor at 2 h time point, and at the 24 h time point, the concentration decreases rapidly. Conclusively, our developed formulation can be accounted for the topical treatment of uveitis, as it exhibited comparable anti-inflammatory activity to the marketed formulation at much-reduced concentration of the drug (0.1%).

## Figures and Tables

**Table 1:** Characteristics of cTA-NLC formulation.

Particle Size (nm)	Zeta Potential (mV)	% EE	pH	Osmolarity (mOsmol/L)
198.9 ± 12.8	35.8 ± 1.9	88.14 ± 3.03	7.21 ± 0.17	298.3 ± 22.7

**Table 2:** TNF-α Elisa assay results of different treatments.

Treatments	TNF-α Values
LPS only	2133.35 ± 283.41
LPS + blank NLC	1573.96 ± 310.02
LPS + TA-solution	1442 ± 312.22
LPS + cTA-NLC	1113.05 ± 55.88

**Table 3:** Total protein count and total cell count of different treated groups.

Groups	Total Protein Count (mg/mL)	Total Inflammatory Cell Count (×10^5^)
Group I	4.95 ± 0.69	52.4 ± 7.71
Group II	1.68 ± 0.23	8.73 ± 1.79
Group III	3.27 ± 0.24	30.13 ± 3.021
Group IV	4.15 ± 0.36	39.46 ± 2.71
Group V	3.70 ± 0.13	13.06 ± 1.52

## Data Availability

The data can be shared upon request.
